# Confirmatory factor analysis of latent constructs for measuring social well-being in African migrant samples

**DOI:** 10.1016/j.heliyon.2024.e29479

**Published:** 2024-04-12

**Authors:** Ufuoma Patience Ejoke, Edwin Devon Du Plessis, Smitha Dev, Ghanem Jaser Al Bustami, Mary Varghese

**Affiliations:** a University of the Free State, Faculty of Humanities, Department of Psychology, Bloemfontein, South Africa; b Abu Dhabi University, Education Department Abu Dhabi, United Arab Emirates

**Keywords:** *African migrants ecosystem*, *Measurement*, *Social well-being*

## Abstract

The present study contributes to psychology and well-being literature by investigating social well-being in minority contexts. The factor structure of the Keyes’ long format 33-item social well-being measure was investigated among African migrant samples. A cross-sectional survey methodology was used to collect data from a total of 404 African migrants living in South Africa (n = 146), Uganda (n = 158) and Kenya (n = 100). They were 202 (50%) males, 195 (48.3%) females, and 7 (1.7%) of the samples did not disclose their gender. They were aged from 14 to 70 with a Mean age of 32.21 (standard deviation = 7.696). Data collected were analysed using CFA in AMOS (version 29). We found an unstable four-factor emic solution for African migrants in Sub-Sahara Africa. We could not replicate the theoretical social well-being model of Keyes (1998) among African migrant samples in Sub-Saharan Africa. Insights from the study will be critical to designing culturally appropriate indigenous measures that accurately reflect the social realities and well-being constructs of African migrants. Our findings will also help policymakers and service providers to identify areas of need, develop appropriate socio-cultural programmes, and allocate resources more effectively to support the integration and well-being of migrants within African societies.

## 1 Introduction

Millions of individuals are relocating across borders in search of better opportunities, safety, and improved quality of life [1]. Among these migrants, those who move within or to Africa represent a diverse group facing unique challenges and opportunities [2]. Understanding their social well-being (SWB) ensures their successful integration into new communities and promotes social cohesion within African societies. Social well-being encompasses various dimensions of individuals’ experiences within their social environment, including their social relationships, sense of belonging, and overall satisfaction with their social circumstances [[3], [4], [5]].

Migration experiences often impact migrants’ SWB profoundly because of the long-term conditions that force them to leave their place of residence [6]; even their relative position as minorities in the societies they have migrated to places them in a position of disadvantage because of their numerical representation, socioeconomic status, cultural differences, or political power. This position accounts for their experiences of segregation or marginalisation from economic activities [7] as well as discrimination in housing, work, educational opportunities, and daily interpersonal interactions [8], which can further impact their SWB. The International Organization for Migration (IOM) asserts that migration exacerbates inequalities that extend across social, cultural, economic, job, and lifestyle aspects [9].

Ejoke [10,11] and Ejoke et al. [2] have shown that migration experience and integration into the new environment have multifaceted implications for African migrants’ adaptation, social health, and well-being.

Psychology, however, neglected the role of worldwide culture and context in understanding wellness and well-being for a long time, accepting that Western perspectives and findings apply globally and across contexts. The skewness of these assumptions is changing in recent times, with voices not only suggesting cultural fair models and indigenous studies [[12], [13], [14]] but the acknowledgment that well-being is situated in an individual micro-space and human lives are shaped by their ecology [15]. These understandings were derived from observable opinions across different regions including the West, East Asia, and Africa [16]. However, insights remain scattered, with the African contexts being neglected, particularly the minority populations. We agree that understanding well-being should be informed by insights from diverse contexts. Well-being is not only characterised by dynamic social processes encompassing material, relational and subjective dimensions [17] but a sense of community belonging and meaningful relationships constitute the essence of social health [17,18]; even these elements play a central role in shaping the very meaning of life for Africans [19]. The role of social and community embeddedness defines Africans [4]. It is implicitly considered an indicator of positive adaptation and well-being of African migrants [2,20,21]. Their promotion has become the central aim of cultural intervention programmes and immigration policies across different countries.

Although there is a growing interest in valid and reliable social well-being measurement instruments in Africa [13,14] and a call for a social well-being scale that is theoretically and contextually driven [22], limited studies on the factorial exploration of the social well-being model for minority populations exist. In line with this need for a contribution, the present study focused on a relatively unexplored topic: investigating the five dimensions of Keyes's [4] social well-being scale (social contribution, social acceptance, social coherence, social actualisation, and social integration) in African migrant samples. The study aimed to achieve a contextually accurate SWB measure for African migrants.

The validation study will play a crucial role in ensuring that measures of SWB are culturally appropriate, valid, and reliable within the African context. Experiences and perceptions of African migrants will also be accurately captured. Researchers will be able to enhance the validity and reliability of their findings, thereby providing a more accurate understanding of SWB among African migrants. Moreover, such a validation will contribute to the development of culturally sensitive interventions. Policies aimed at promoting the SWB of migrants in Africa will be able to identify and address the unique challenges African migrants face. Policymakers and service providers can better support their integration, well-being, and social inclusion within diverse communities across the continent.

## 2 Social well-being and African migrants

The inquiry into social well-being has raised important questions about its underlying structure. Keyes [4] defined SWB as a person's self-assessment of the relationship quality they experience with others, their connection to their neighbourhood, and their sense of belonging within the broader community. This unique perspective on social well-being distinguishes itself from existing measures that typically examine either interpersonal aspects (such as social support) or societal factors (including poverty and social capital) at a macro level [23]. Keyes's conceptualisation of social well-being encompasses various elements that collectively reflect an individual's level of functioning within their social context, whether as neighbours, citizens, or migrants in a new environment [4]. Keyes [4] operationalised and validated social well-being from a health model framework by identifying five key dimensions. These dimensions include individuals' perceptions of their level of integration into society (social integration), their sense of acceptance by others (social acceptance), their perception of the coherence of social structures and events (social coherence), their feeling of contributing meaningfully to society (social contribution) and their perception of the potential for growth and development within society (social actualisation).

Consequently, our study specifically focused on determining whether this five-factor social well-being measurement, commonly applied in Western contexts and recently validated in Africa [13], can be adapted effectively for use in a minority group such as the migrant population. This adaptation becomes particularly relevant when considering the unique experiences and perspectives of African migrants.

During the last two decades in South Africa, tensions have arisen between Black South Africans and African migrants. African migrants are not only accused of taking South African women and stealing jobs that belong to locals but also of running thriving drug trades within townships. Recently, these narratives were strongly re-echoed by Operation Dudula, an anti-migrant group in South Africa, whose mandate is to eradicate migrants from South Africa; “Dudula” means “push out” in the Zulu language [24].

The winds of hostility are also blowing strong in Kenya and Uganda. In Kenya, xenophobia is on the increase; the Kenyan military constructed a razor-wire fence that runs along the entire 440-mile border between Kenya and Somalia [25]. The Somali community in North-Eastern Kenya is stigmatised and accused of crimes and acts related to terrorism, which damages the reputation of all Somali natives residing in Kenya. Xenophobia mocks social integration, and trade relations between Somalis and Kenyans are one-sided. For example, the Somalis would rent out their properties to Kenyans, but many Kenyans would not let the Somalis use their properties [7].

In Uganda, Gagliardi [26] analysed migrants’ problems, using a variety of methodologies, including "cultural consonance," "ethnic density," and "socio-cognitive niche" and found that migrants frequently struggle to obtain mental health treatment. The reasons included communication difficulties with mental health professionals, legal obstacles, financial difficulties, and taboos surrounding mental health concerns. Prejudices towards immigrants or minorities have also been linked to diagnostic errors, since individuals without schizophrenia may be diagnosed with the illness.

Apart from that, migrants in Uganda were found to be more vulnerable to low dietary diversity compared to locals [27].

While the continuous rejections, hostilities, and mistrust from locals are social challenges within the ecosystem that define migrants’ lives and well-being [2], separation from their families, the loss of connection to their home culture or meaning of home is a notable issue which further leads to feelings of detachment and cultural disorientation [21]. Disruptions to social and emotional support networks and means of subsistence [28], unanticipated difficulties adjusting to new surroundings [29], uncertainty about immigration status [[30], [31], [32]], the struggle to achieve their personal goals and fulfill basic needs, or inability to engage in meaningful daily activities, loss of control over their living conditions and destiny contributes to a sense of frustration and dissatisfaction. Their ability to play a meaningful role within their new community is further compromised, potentially leading to feelings of exclusion and isolation [11,20].

Over time, the cumulative impact of these challenges, such as the loss of connection to their home culture or the rejection from locals and the ongoing process of adapting to the host culture, can take a toll on the well-being of African migrants [2,33]. Consequently, scholars focusing on well-being in African contexts should not only consider well-being from the domains of social, relational, and communal [4,34,35] but also consider the situatedness of human beings within their eco-space [15], particularly African migrants, within their unique socio-structural and community context.

Unfortunately, little is known about social well-being among migrant populations. We know that the nature of a well-lived life embraces life into public tasks and private tasks. Attaining positive social health is associated with the social dimension of life, with life challenges serving as criteria for judging the quality of their lives [4]. We also know that large-scale representative studies, numerous small studies, and several world reports have suggested that social well-being is necessary to conceptualise and measure well-being [9,36,37]. Social well-being scales have also been validated across different countries in multiple environments and cultures [13,22,[38], [39], [40], [41]].

These validations, however, remain limited for use in minority contexts; the few studies found were primarily derived from native samples in their countries of birth. For example, Albrithen [38] validated the 15-item SWBS for a sample of Emiratis and found the scale accuracy for measuring SWB in Arab culture. Li et al. [39] also replicated the scale in Chinese samples and Silva et al. [41] confirmed it for Portuguese samples. Shayeghian et al. [40] only retained the intended factor structure in Iranian samples after minor modifications in three of the dimensions (i.e. social integration, social coherence, and social acceptance).

Interestingly, we did not find notable psychometric studies on social well-being in Kenya and Uganda. However, two notable studies with contextual and meaningful interpretations of the factor solution to our exploration were found in South Africa – Khumalo et al. [13] and Jager et al. [22]. Khumalo et al. [13] investigated the factor structure of the 15-item social well-being scale among university student samples and found an unstable emic four-factor solution. Jager et al. [22] were also unable to replicate Keyes's [4] theoretically intended model in South African motor employee samples. They found a three-factor solution. These inconsistencies of prior studies results have led to concerns about operationalizing social well-being differently across countries, particularly in South Africa [22]. Even the recommendation of validating the long-form of the social well-being scale for better factorial stability [13].

Notably, the validation of the short form of social well-being of Keyes [4] is commonly used in native samples. However, using the 33-item long format scale, Lages et al. [42] replicated Keyes’ theoretically five-factor structure for Portuguese samples. Their study yielded good concurrent validity. Validating the 33-item scale in a minority African context is critical to designing a contextually relevant minority well-being intervention. Khumalo et al. [13] carefully expressed this concern when they found an unstable four-emic structure in Africa. This necessitated their suggestion for future studies exploration of the long- and short-form SWB in different African samples. The inconsistent findings in factor structures observed in prior studies underscore the need for measuring and interpreting social well-being among migrant populations, not only because of their cultural variations but also due to the complexity of migrant lives while in their new cultures. The concern of operationalizing SWB in South Africa [22] highlights the importance of culturally sensitive measurement of social well-being. This study addressed the gaps by validating the latent construct of the 33-item social well-being scale of Keyes [4] for African migrant samples in Sub-Saharan Africa.

## 3 Present study

The present study extended prior empirical investigations by Lages et al. [42] and others. We explored the factor structure of Keyes's [4] 33-item Social Well-being Scale using confirmatory factor analysis (CFA) focusing not only on African samples but also on migrant populations across three countries (South Africa, Uganda, and Kenya). This broader scope allowed us to explore the applicability of the social well-being model in diverse cultural and geographical contexts. A comprehensive understanding of mental health is contingent upon the availability of assessment tools that are both theoretically valid and appropriate for the contexts in which they are used [42]. Building on this perspective, Khumalo et al. [13] noted the inconclusive findings in prior research and recommended future studies to use the long-form Social Well-Being Scale (SWBS) for deeper insights into the scale factorial stability. To contribute to the field, our study addresses the observable research gap by exploring the long-form of SWBS to determine whether the five indicators of Keyes's social well-being, i.e. social integration, social contribution, social coherence, social actualisation, and social acceptance remain relevant and applicable for measuring social well-being in African populations, particularly, African migrants. Through the exploration of the factor structure of these indicators in the context of our heterogeneous sample, we sought to provide valuable insights into the suitability of Keyes's model for understanding social well-being in Sub-Saharan African migrant populations.

## 4 Methods

### 4.1 Participants and settings

Data for this study was collected from a total of 404 African migrants through the cross-sectional survey method. Respondents were aged from 14 to 70 with a mean age of 32.21 years (s.d. = 7.696). The data was collected in 2022. Sample demographic characteristics are presented in Table 1.

**Table 1:** Demographic characteristics of the sample (n = 404).

Variable	Level	N	%
Host countries	South Africa	146	36.1
	Uganda	158	39.1
	Kenya	100	24.8
Gender	Males	202	50
	Female	195	48.3
	Undisclosed gender	7	1.7
Age, years	14–70	403	–
Educational level	No education	14	3.5
	Primary	111	27.5
	Secondary	94	23.3
	Diploma	78	19.3
	BSc	47	11.6
	Postgraduate	60	14.9

The snowball sampling technique was used to locate and recruit African migrants in South Africa. Data were collected in the following communities and settings: Rhodesfield, Pretoria, UNISA, T.U.T., Springs, Johannesburg CBD, Turffontein, and Soweto. In Uganda, African migrants were conveniently recruited at the UNCHR offices and Bukesa Village in Central Kampala, where data were also collected. Using the same convenient sampling in Kenya, data were collected in Nairobi City County – Kawangware, Jamhuri, Pangani, and Kasarani, as well as in Kajiado county around Kitengela town and Umoja Refugee Organisation CBO (Community-Based Organisation) in Kawangware.

## 5 Measuring instrument

### 5.1 Social well-being scale long-form

Keyes's [4] Social Well-Being 33-item long form measures social well-being on five dimensions that show how people evaluate their social functioning and surroundings. The response format is a 6-point Likert scale, with a range of 1–6 representing strongly disagree (1) to strongly agree (6). The social integration dimension contains seven elements, the social contribution dimension has six, the social coherence dimension has six, the social actualisation dimension has seven, and the social acceptance dimension has seven. Lages et al. [42] observed the following: Social Acceptance (α = 0.86), Social Actualisation (α = 0.76), Social Integration (α = 0.80), Social Contribution (α = 0.70), and Social Coherence (α = 0.67). They also discovered that the lengthy version of the SWBS was trustworthy among the Portuguese samples.

### 5.2 Ethics declarations

Data for the present study emerged from a bigger funded project named Meaning-making and Sources of Meaning as Pathways to Well-being and Positive Adjustment among African migrants in selected Sub-Saharan countries. A grant from the 10.13039/501100001321National Research Foundation (NRF) (number 129662) was used to collect data for the current study after the 10.13039/501100004765University of the Free State Research Ethics Committee granted ethical approval (number UFS-HSD2020/2142/223/21).

The ethical principles were followed throughout the data collection process in all three countries (South Africa, Uganda, and Kenya). Participants only provided their informed consent after they had fully understood the goal of the study. They were also informed of their voluntarism in participation and withdrawal at any time should they feel uncomfortable with their participation.

### 5.3 Data analysis

The present study used CFA in AMOS to investigate the model fit of the 33-item SWS-LF. The five-factor model was tested with CFA using both the oblique geomin rotation and robust maximum likelihood (MLR) estimate. We tested the model fits with chi-square (χ2), standardised root mean square residual (SRMR), Akaike information criterion (AIC), Bayesian information criterion (BIC), comparative fit index (CFI), and root mean square error of approximation (RMSEA) [43]. To establish a good fit, the following standards were applied: smaller and insignificant χ2, RMSEA of less than 0.06, CFI of greater than 0.95, GFI of greater than 0.90, and lower AIC and smaller BIC [44,45].

## 6 Results

### 6.1 Descriptive analysis

The skewness values (skewness <3; kurtosis <8) indicated that the assumption of normality was satisfied [46]. Except for two questions (skewness, item 20) and kurtosis, item 4, most of the items indicate that respondents gave answers in the range of one to six, suggesting that our items were relatively well-discriminated as shown in Table 2. Moreover, the absence of any reported missing values enhances the data quality and the degree of dependability of the findings.

**Table 2:** Descriptive statistics: Means, Standard deviation, Skewness and Kurtosis.

Descriptive Statistics									
Items						Skewness		Kurtosis	
	N	Min	Max	Mean	Std dev	Statis-tic	Std. Error	Statis-tic	Std. Error
S1	404	1	6	3,67	1985	-,235	,121	−1557	,242
S2	404	1	6	4,82	1247	−1200	,121	1128	,242
S3	404	1	6	4,64	1277	−1059	,121	,752	,242
S4	404	1	9	4,71	1269	-,950	,121	,925	,242
S5	404	1	6	4,52	1413	-,952	,121	,150	,242
S6	404	1	6	3,59	1652	-,205	,121	−1140	,242
S7	404	1	6	4,62	1251	−1009	,121	,797	,242
S8	404	1	6	4,22	1570	-,658	,121	-,574	,242
S9	404	1	6	4,08	1502	-,546	,121	-,489	,242
S10	404	1	6	4,52	1385	-,859	,121	,061	,242
S11	404	1	6	4,40	1520	-,720	,121	-,440	,242
S12	404	1	6	4,60	1397	-,854	,121	-,030	,242
S13	404	1	6	4,55	1459	-,843	,121	-,161	,242
S14	404	1	6	3,79	1737	-,302	,121	−1160	,242
S15	404	1	6	5,09	1270	−1767	,121	2839	,242
S16	404	1	6	5,21	1131	−1826	,121	3562	,242
S17	404	1	6	3,07	1769	,197	,121	−1358	,242
S18	404	1	6	3,08	1801	,269	,121	−1334	,242
S19	404	1	6	4,82	1355	−1299	,121	1081	,242
S20	404	1	6	2,84	1845	,475	,121	−1273	,242
S21	404	1	6	3,51	1703	-,104	,121	−1266	,242
S22	404	1	6	3,65	1655	-,267	,121	−1093	,242
S23	404	1	6	4,01	1677	-,558	,121	-,950	,242
S24	404	1	6	4,70	1353	−1005	,121	,396	,242
S26	404	1	6	3,68	1708	-,171	,121	−1183	,242
S27	404	1	6	3,62	1674	-,238	,121	−1101	,242
S28	404	1	6	4,06	1705	-,433	,121	−1055	,242
S29	404	1	6	3,38	2006	,065	,121	−1624	,242
S30	404	1	6	3,84	1817	-,279	,121	−1340	,242
S31	404	1	6	4,37	1514	-,767	,121	-,404	,242
S32	404	1	6	4,89	1328	−1202	,121	,701	,242
S33	404	1	6	4,50	1705	-,828	,121	-,647	,242

**Fig. 1:**
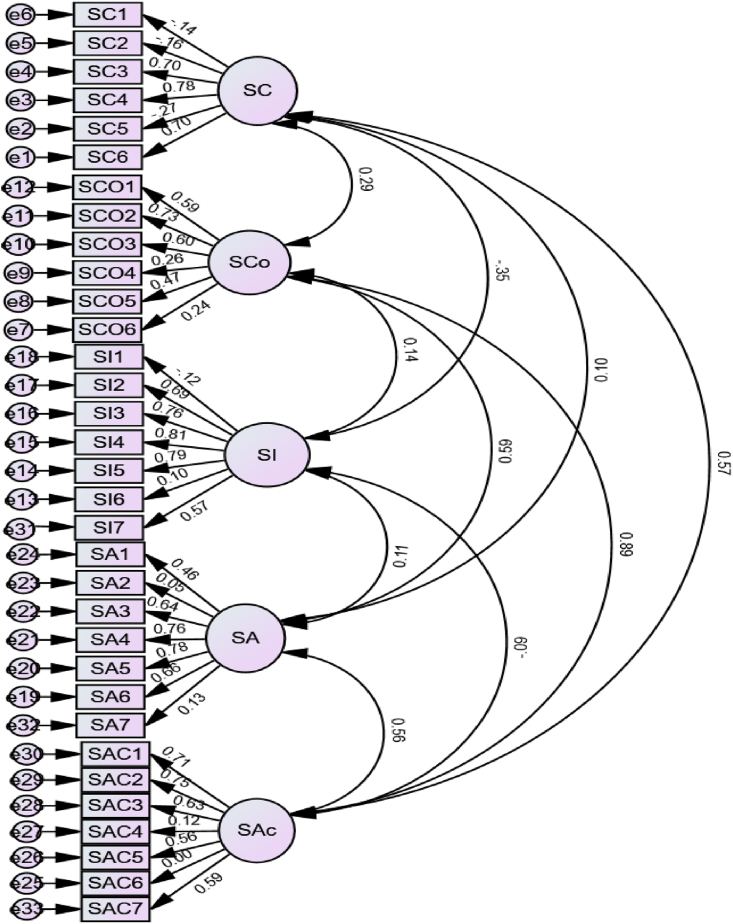
Confirmatory factor analysis of the raw model.

**Fig. 2:**
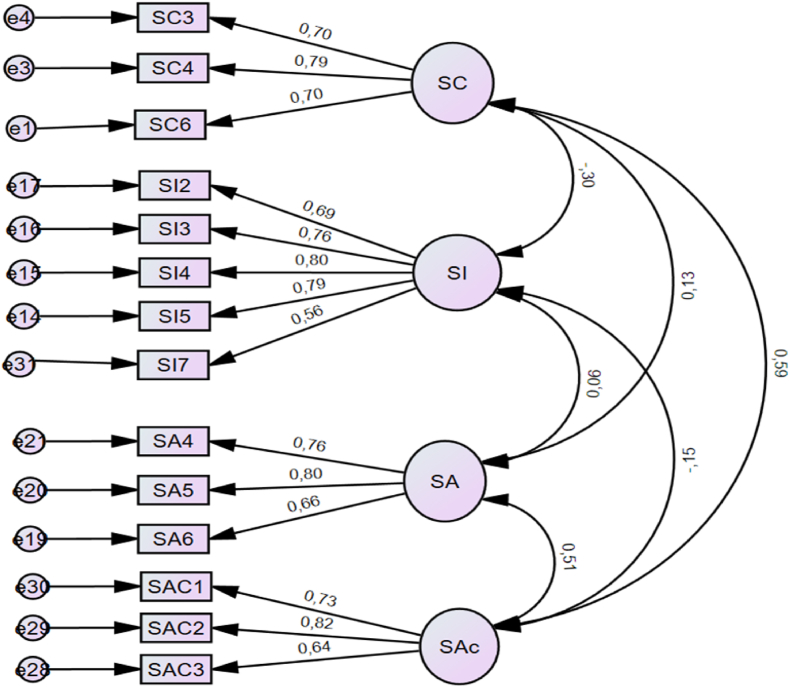
Improved model from the Confirmatory Factor Analysis.

The initial theoretical and measurement model (Fig. 1 also known as study Model 1), consisting of 33 items, was taken into consideration when performing the confirmatory factor analysis. This was done by using the Unit Variance Identification (UVI) strategy, which allows us to compare estimates for each dimension. However, this first model yielded poor fit indices, χ2 (48) = 3.162, p < 0.000; CFI = 0.740; RMSEA = 0.73, p < 0.000 [0.069 0.077]. With a χ2 (71) = 1.838, p < 0.000, CFI = 0.971, RMSEA = 0.046, p = 0.710 [0.033 0.058], Model 2 fits the data better (See Fig. 2).

Table 3 presents the model fit indices.

**Table 3:** Model fit indices.

	χ2	df	p	AIC	BIC	RMSEA	RMSEA 90% CI		CFI	GFI
							Lower	Upper		
**Model 1**	3.162	48	0.000	1685.396	1989.503	0.073	0.069	0.077	0.740	0.791
**Model 2**	1.838	71	0.000	226 505	230 217	0.046 0.71	0.033	0.058	0.971	0.956

We removed items that were not valid from the raw model to improve the model. We used the standardised residual co-variance to identify these items, and all the trends of numbers that significantly deviated from 2.4 in absolute value were removed. The social coherence dimension was totally removed from the model; the dimensions of social contribution, social acceptance, and social actualisation had only three of their items, each with factor loadings above 0.30. Then, social integration had five out of its seven items with factor loading above 0.30. Only the items with a factor loading greater than 0.30 are shown in Table 4. Keyes's [4] five-dimensional structure is untenable for African migrants because of its unstable internal inconsistency.

**Table 4:** Confirmatory Factor Analysis Standardised Regression Weights for African migrants in Sub-Sahara.

			Estimate	P value
Latent and Indicator variables				
Social contribution				
SC6	<---	SC	0.701	<0.001
SC4	<---	SC	0.789	<0.001
SI5	<---	SI	0.791	<0.001
SI4	<---	SI	0.803	<0.001
SI3	<---	SI	0.764	<0.001
SI2	<---	SI	0.694	<0.001
SA6	<---	SA	0.661	<0.001
SA5	<---	SA	0.798	<0.001
SAC3	<---	SAc	0.640	<0.001
SAC2	<---	SAc	0.817	<0.001
SAC1	<---	SAc	0.730	<0.001
SI7	<---	SI	0.561	<0.001
SC3	<---	SC	0.702	<0.001
SA4	<---	SA	0.762	<0.001

### 6.2 Construct reliability and convergent validity

Further examination of the construct reliability and convergent validity is contained in Table 4. The construct reliability (internal consistency) of the final model was good and appropriate as they were all greater than 0.70: social actualisation (α = 0.77), social acceptance (α = 0.78), Social integration (α = 0.85), and social contribution (α = 0.77). The Convergent Validity (AVE) was also greater than 0.50, Table 5 shows that AVEs for social integration, social contribution, social acceptance, and social actualisation are 0.551, 0.536, 0.530 and 0.537, respectively.

**Table 5:** Reliability of final model.

Construct	N	Mean	Std. Deviation	CR (Reliability >0.7)	AVE (Convergent Validity >0.5)
Social integration	404	23.32	25.77	0.85	0.551
Social contribution	404	8.99	4.4.90	0.77	0.536
Social acceptance	404	13.55	13.35	0.78	0.530
Social actualisation	404	11.17	17.47	0.77	0.537

### 6.3 Discriminant validity

The discriminant validity of the final model was established. The values of the variables were adequate when considered against their squared correlation, as indicated in Table 6.

**Table 6:** Discriminant validity: Fornell and Larker (1981) matrix.

	SA	SC	SI	SAc
**SA**	**0.743**			
**SC**	0.133	**0.732**		
**SI**	0.060	−0.302	**0.728**	
**Sac**	0.513	0.589	−0.152	**0.733**

The square roots of social acceptance (SA) were 0.743, comparing this value with the column, whereby all the values (0.133, 0.060, 0.513) under this column were below the square root of 0.743. Thus, the discriminant validity of the social acceptance construct was established. The same applied to the square roots of SC (0.732), SI (0.728), and SAc (0.733), which were above the correlation of other constructs.

## 7 Discussion

The study aimed to achieve a contextual and accurate SWB measure for African migrants. The 33-item social well-being scale was validated for African migrant samples living in Sub-Saharan Africa, and structural equation modelling showed a deviation from Keyes's [4] theoretical five-factor model. The model yielded a poor fit. However, after modification of the original model, new interpretable four dimensions emerged with acceptable internal consistencies. The dimensions were community engagement and service (three items), social recognition and belonging (five items), social selflessness and trust (three items), and sustainable support system (three items). We observed that from the 33 items, only 14 items remained relevant for the African migrant population. None of the six items on the social coherence dimension were retained.

Items were also removed from the other dimensions. For example, two items were removed from social integration: ‘*You don't feel you belong to anything you'd call a community’; ‘If you had something to say, you don't think your community would take you seriously’*. Items removed from social acceptance include: ‘*You think that other people are unreliable’; ‘You believe that people are kind’; ‘You believe that people are self-centered’; ‘You think that people care about other people's problems'.* Items excluded from social contribution were ‘*Your behaviour has some impact on other people in your community’, ‘You think you have something valuable to give to the world’, and ‘You think that your work provides an important product for society’.* Items removed from social actualisation included ‘*You see society as continually evolving’; ‘You think our society is a productive place for people to live in’; ‘For you, there's no such thing as social progress’; ‘You think the world is becoming a better place for everyone’*.

A closer look at the removed items presented contextual interpretation, offering reasons for their exclusion. Three possibilities may explain why these items did not resonate with African migrant samples. The first is linked to sample specificity. The challenges of immigration, particularly the loss of connection to migrants' home culture or the daily task of adapting to the host culture, can be exhausting over time for African migrants [33]. This may have contributed to their inability to appraise or predict their host community. The second possibility may be connected with the push factors from their homelands, which may not only challenge migrants’ identity and meaning systems but may offer possibilities for them to develop resilience, which may affect their beliefs about the world and ability to predict future goals [47].

Third, the insistence of the items on citizens' sense of value or worth and institutional system expected support is another possible explanation for item removal. Migrants’ ability to relate to the meaning of these items might have been distorted because their ability to retain and enhance perceptions of control and stability is compromised during their trajectory experience [2]. Migration in itself has implications for how migrants perceive the world and circumstances around them. Their inherent assumptions and views on the benevolence of the world, the meaningfulness of the world, and self-worthiness tend to be broken due to their unpleasant experiences [48].

Janoff-Bulma [48] theorised through shattered assumptions that assumptions are broken when people experience traumatic events. The challenging events within the migration journey make the assumption of communities’ tolerance and support unpredictable for migrant populations whose future seems uncertain. The uncertainty experienced at homelands with continuous relationality and material inequality experiences in host countries imply the complexity and senselessness of the surrounding worlds for the majority of African migrants. It is possible, as argued by Mirowsky and Ross [49] and Seeman [50,51], that the situatedness of people shapes their being. African migrants may attribute meaninglessness to circumstances surrounding their lives or become uncertain and unpredictable because of their migration and adaptation experiences.

## 8 Community engagement and service (three items)

Community engagement and service comprised the salient loading of three items, namely: *Item 3: ‘Your daily activities do not produce anything worthwhile for your community’; Item 4: ‘You don't have the time or energy to give anything to your community’, and Item 6: ‘You feel you have nothing important to contribute to society’*. These items were indicated by the social contribution of Keyes's Social Well-Being Scale. Community engagement or a sense of service given to the community has implications for developing social ties with neighbours, gaining a sense of connectedness, and strengthening social identity [52]. These factors are crucial for African migrants' adaptation and daily living in the host environment [2].

Participation in the community is a sign of people's well-being as well as life aspirations [53]. Community engagement in community psychology refers to the idea that emotionally attached individuals live extra-individually in healthy communities, and this idea is typically expressed in individual and collective lives [54,55]. Community engagement is therefore considered vital for orienting interventions to increase individual well-being within the communities [56]. This is why the Sense of Community (SoC) model put forth by McMillan and Chavis [57] includes dimensions including membership, influence, integration, meeting needs, and shared emotional connection.

## 9 Social recognition and belonging (five items)

Social recognition and belonging contain the salient loading of four items: *Item 2: ‘You feel like you're an important part of your community’; Item 3: ‘If you had something to say, you believe people in your community would listen to you’; Item 4: ‘You feel close to other people in your community’; Item 5: ‘You see your community as a source of comfort’, and Item 7: ‘You believe other people in society value you as a person’,* formerly included within the social integration dimension of Keyes. In a study conducted by Khumalo et al. [13], Item 5 was considered to represent the community as a source of safety, while De Jager et al. [22] represented it within the dimension of social trust. Although these studies used African samples, the different interpretations of the same item indicate different operationalisation, even for minority groups. This also suggests that the remaining items will present different meanings and interpretations for African migrant samples.

## 10 Social selflessness and trust (three items)

The social selflessness and trust dimension consists of items 4, 5, and 6. *Item 4: ‘You feel that people are not trustworthy’. Item 5: ‘You think that people live only for themselves’, Item 6: ‘You believe that people are more and more dishonest these days’.* These items were included in Keyes's social acceptance dimension. These three items speak to the strength of oneness [58,59], which exudes kindness, loyalty, honesty, generosity, and caring. Sense of caring, compassion, knowledge, generativity towards others, and perceptions of civic responsibilities were positively connected to overall social well-being [60]. A person's perception of neighbourhood safety and neighbours' trustworthiness is important in social well-being [4]. This is because it enhances place attachment. Evidence has shown that understanding well-being requires an understanding of one's relationship to one's living environment [61], as the sense of well-being, quality of life, and the perception of one's living environment as well as feeling at home in one's neighbourhood are intertwined [[62], [63], [64]].

## 11 Sustainable support system (three items)

The items that form a sustainable support system for the migrant sample were items 1, 2, and 3. *Item 1: ‘You believe that society has stopped making progress’, Item 2: ‘Society isn't improving for people like you’, and Item 3: ‘You don't think social institutions like law and government make your life better’.* These were among other items in the social actualisation dimension in Keyes's SWBS. In the study of Khumalo et al. [13], Item 2 was indicated as part of the world as an understandable dimension. In a scoping review on meaning-making for migrants' well-being and positive adjustment conducted by Ejoke et al. [2], social networks and support communities rank among the most popular coping mechanisms utilized by immigrant populations. The review focused on meaning-making for migrants' well-being and good adjustment. The ease of use and efficacy of social networks might impact an individual's capacity to cope with their present circumstances as well as potentially traumatic prior experiences. Social assistance can be given by neighbours, family, close friends, and other locals. In addition to more obvious types of practical assistance like food or shelter, among other things, this support may help the person make sense of their situation or feel less alone [65,66].

Sustainable support systems are beneficial for the migrant population. Systemic support will ease stressors related to displacement, health, loss of loved ones, and day-to-day survival difficulties including domestic violence [66,67]), as well as perceived discrimination [68].

### 11.1 Limitation and recommendations

While it may appear from this study that the long-form of the Social Well-Being Scale is a more appropriate scale version to provide more insight into the factorial stability of the SWB model [13], the inconclusive findings encourage future studies in diverse samples, particularly in minority populations. These studies should ensure the inclusion of more diverse African migrant samples. Qualitative studies that delve into a particular subject's nuances, meanings, and perspectives may help to contextualize the social well-being of the African migrant population.

## 12 Conclusion

The present study appears to be the only one that has looked into and validated social well-being among African migrant samples, despite acknowledging earlier empirical investigations that have used African samples [13,22] whose findings also confirm the heterogeneity and factorial instability of the model. Through a confirmatory factor analysis, we found that the conventional five-dimensional structure of Keyes's model does not hold among African migrant samples in Sub-Saharan Africa. Instead, an unstable four-factor emic solution emerged, attesting to the multidimensional interpretation and understanding of well-being across cultures and among minority groups. Our findings suggest the need for a contextual and culturally nuanced understanding of SWB among these populations, while also confirming the role of the eco-space and relationality in shaping human well-being [15].

Our study presents multifaceted implications, reflecting academic research, policy development, and social intervention strategies. Our findings challenge the universality of Western models of SWB, highlighting the critical role of cultural and contextual factors in shaping the constructs of well-being. This necessitates a pivot towards developing indigenous models and scales that accurately reflect the social realities and well-being constructs of African migrants. For academia, this underscores the importance of incorporating cultural competence in research methodologies and the development of theoretical frameworks that are inclusive and representative of diverse populations.

The study's outcomes signal a clear need for more targeted and culturally sensitive policies and programs that address the unique challenges and needs of African migrants. Understanding the distinct dimensions of their social well-being can guide policymakers and service providers in creating more effective integration and support mechanisms, which are vital for promoting social cohesion and the overall well-being of migrants. For service providers working directly with migrant communities, the study offers insights into the social well-being dynamics at play. It suggests that interventions aimed at enhancing migrants' social well-being should consider the cultural specificity and relevance of their strategies, ensuring that these are grounded in the lived experiences and social realities of African migrants.

The present study thus contributes to a growing body of knowledge that seeks to redefine and contextualize social well-being in African migrant populations. It calls for a concerted effort among researchers, policymakers, and practitioners to embrace a more nuanced understanding of well-being that respects cultural differences and promotes the development of supportive environments for migrants. By doing so, we can better address the complexities of migration and integration, fostering societies that are more inclusive and attuned to the diverse needs of their members.

## Funding information

This study is funded by the 10.13039/501100001321National Research Foundation (NRF) with a grant [number 129662]. Funding institutions might not always be involved in the creation of content.

## Data availability

A cross-sectional survey of African immigrants residing in South Africa, Uganda, and Kenya provided the study's data. Upon request, the data set will be made available.

## Disclaimer

The views expressed in this article are those of the authors alone, and they have no bearing on any official national policy or stance taken by any of the authors' connected agencies.

## CRediT authorship contribution statement

**Ufuoma Patience Ejoke:** Writing – review & editing, Writing – original draft, Methodology, Funding acquisition, Data curation, Conceptualization. **Edwin Devon Du Plessis:** Conceptualization. **Smitha Dev:** Writing – review & editing, Conceptualization. **Ghanem Jaser Al Bustami:** Writing – review & editing, Conceptualization. **Mary Varghese:** Writing – review & editing, Conceptualization.

## Declaration of competing interest

The authors declare no conflict of interest in this study titled Confirmatory Factor Analysis of Latent Constructs for Measuring Social Well-being in African Migrant Samples. The authors declare that they have no financial or personal relationships that may have inappropriately influenced them in writing this article.
